# Long-term Outcomes of a Contemporary Arthroscopic Bankart Repair Technique in Patients With Traumatic Anterior Shoulder Instability: A Minimum 10-Year Follow-up

**DOI:** 10.1177/03635465251351293

**Published:** 2025-07-07

**Authors:** Anh Do, Markus Scheibel, Philipp Moroder, Agahan Hayta, Isil Akgun Demir, Alp Paksoy, Doruk Akgün

**Affiliations:** † Center for Musculoskeletal Surgery, Charité–Universitätsmedizin Berlin, Berlin, Germany; ‡ Department of Shoulder and Elbow Surgery, Schulthess Klinik, Zurich, Switzerland; § Department of Plastic, Reconstructive and Aesthetic Surgery, Istanbul Atlas University, Istanbul, Turkey; Investigation performed at the Center for Musculoskeletal Surgery, Charité–Universitätsmedizin Berlin, Berlin, Germany

**Keywords:** Bankart repair, arthroscopic surgery, recurrence rate, shoulder instability

## Abstract

**Background:**

The long-term results of arthroscopic Bankart repair are poorly reported in the literature and show recurrence rates as high as 37%. However, this high failure rate is based on historical studies of patients with critical defects stabilized using older techniques.

**Purpose:**

To evaluate the long-term clinical outcomes of arthroscopic Bankart repair using a contemporary technique with a minimum of 3 suture anchors in patients with traumatic anterior instability and to assess possible risk factors for recurrent instability.

**Study Design:**

Case series; Level of evidence, 4.

**Methods:**

A total of 181 patients (182 shoulders), who underwent arthroscopic Bankart repair because of traumatic anterior instability between 2005 and 2014, were included in this study and evaluated at a minimum follow-up of 10 years. Exclusion criteria were previous shoulder surgery, additional stabilization procedures, use of <3 anchors, multidirectional instability, and indications for bony reconstruction (critical bony defects: glenoid defect >20%, off-track Hill-Sachs lesion). The primary outcome was recurrent instability. Secondary outcomes were the Subjective Shoulder Value (SSV), visual analog scale (VAS), Constant score, Western Ontario Shoulder Instability Index (WOSI), and Rowe score as well as sports activity level and return to sports. Risk factors for recurrent instability were analyzed.

**Results:**

The overall recurrence rate was 20.9% at a mean follow-up of 13.8 ± 2.8 years. Age ≤20 years at the time of surgery was associated with a higher risk of recurrence (*P* = .007). The failure rate was lower in patients who underwent surgery after the first-time dislocation (8/58 [13.8%]) compared with patients who underwent surgery after multiple instability events (30/124 [24.2%]), although this was not statistically significant (*P* = .108). Patients without recurrent instability had statistically significant better scores on the SSV (*P* < .001), VAS for pain during movements (*P* = .016), Constant score (*P* = .011), WOSI (*P* = .001), and Rowe score (*P* < .001) compared with patients with recurrence without revision surgery. A shorter interval between the first dislocation and surgery was associated with better shoulder outcomes, despite a consistent recurrence rate. Of all patients, 97.6% returned to sports, with 69.6% returning to 90% to 100% of their preoperative sports activity.

**Conclusion:**

Arthroscopic Bankart repair resulted in a relatively high recurrence rate, despite the use of a contemporary technique, particularly in patients with >1 dislocation before surgery. While younger age and a higher number of preoperative dislocations were potential risk factors for recurrence, a shorter interval between the first dislocation and surgery was associated with improved clinical outcomes. Therefore, patients with a high risk of redislocations should be considered for early soft tissue stabilization, while additional procedures such as remplissage should be performed for those with nonmodifiable high-risk factors.

An anteroinferior shoulder dislocation is one of the most common shoulder injuries and typically results in detachment of the capsulolabral complex from the anterior glenoid rim (Bankart lesion) as well as an impression fracture of the humeral head (Hill-Sachs lesion).^3^ Both can lead to recurrent shoulder instability; thus, surgical stabilization is recommended, particularly in young, active patients with a high risk of redislocations.^16,18^ Risk factors include young age, male sex, participation in overhead or collision sports, involvement in sports at a high competitive level, hyperlaxity, and bony lesions.^2^

Long-term results with a minimum follow-up of 10 years after arthroscopic Bankart repair are rarely reported and show recurrence rates as high as 37%.^1,9^ However, the high failure rate may primarily be attributed to previous studies using older techniques, such as transglenoid sutures^38^ or bioabsorbable tacks,^1,19,26,27^ and <3 anchors.^1,7,10,33,34^ Therefore, these studies may not represent the results using contemporary surgical techniques with at least 3 suture anchors.^15,22^ Moreover, most studies have included patients who were not selected according to current standards regarding bony defects. This may alter the results, as the significance of these defects in recurrent instability and concepts such as the glenoid track described by Itoi et al^17^ were not widely recognized in the past.

Therefore, the purpose of the present study was to evaluate the long-term clinical results after arthroscopic Bankart repair using a standardized contemporary technique with at least 3 suture anchors in appropriately selected patients with no significant bone loss. Furthermore, the risk factors for recurrence after Bankart repair were analyzed.

## Methods

### Study Cohort

**Figure 1.**
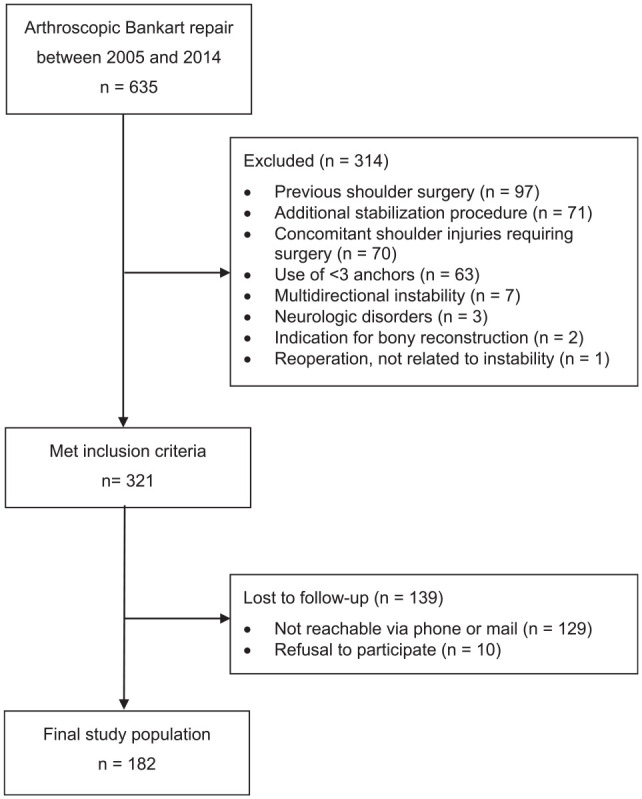
Flowchart of patient enrollment.

For this study, we included 635 patients with traumatic anteroinferior shoulder instability who underwent arthroscopic Bankart repair using a minimum of 3 suture anchors between 2005 and 2014 at our institution. Exclusion criteria were previous ipsilateral shoulder injuries and surgery, additional stabilization methods such as remplissage or capsular plication, concomitant shoulder injuries requiring surgery such as rotator cuff tears or SLAP (superior labrum anterior and posterior) lesions, the use of <3 anchors, multidirectional instability, neurological disorders such as epilepsy, indications for bony reconstruction (critical bony defects: glenoid defect >20%, off-track Hill-Sachs lesion), and reoperations in the same shoulder not related to the instability problem (eg, acromioclavicular joint stabilization). Of 321 shoulders who met the eligibility criteria, 182 shoulders (181 patients) were available and evaluated at a minimum follow-up of 10 years (Figure 1). The study was approved by the local ethics committee (EA4/240/23), and all patients provided written informed consent to participate in the study.

### Surgical Technique and Postoperative Rehabilitation

All surgical procedures were performed by 6 surgeons at a single institution. Under general anesthesia, the patient was placed in the lateral decubitus position, and a diagnostic arthroscopic examination was performed through a standard posterior portal to assess any concomitant shoulder injuries. In all patients, an anteroinferior capsulolabral lesion was evident. After additional anteroinferior and anterosuperior portals were established, the capsulolabral complex was mobilized, and the glenoid rim was freshened.

At our department, 4 different suture anchor systems were used sequentially: first the Bio-FASTak (26.0% of shoulders; hard-body anchor [Arthrex]), the PushLock (45.0% of shoulders; hard-body anchor [Arthrex]), then the Lupine (14.8% of shoulders; hard-body anchor [DePuy Mitek]), and finally the JuggerKnot (9.3% of shoulders; all-suture anchor [Zimmer Biomet]). The Bio-FASTak, Lupine, and JuggerKnot suture anchors were used with the anchor-first technique, and the PushLock suture anchor was used with the knotless suture-first technique. In 9 shoulders, information regarding the type of suture anchors used was unavailable.

For the anchor-first technique, the first suture anchor was placed into a prepared drill hole in the anterior glenoid neck at the 5:30 clock position. Both sutures were shuttled through the anterior capsulolabral complex from inferior to superior to form a mattress stitch, reattaching the labrum to the glenoid neck and creating a capsular shift. These steps were repeated with a minimum of 3 anchors.

For the suture-first technique, a SutureLasso (Arthrex) was passed through the capsulolabral complex, and a FiberWire suture (Arthrex) was shuttled through, forming a cinch stitch. The sutures were inserted with an anchor placed into a prepared drill hole in the anterior glenoid neck to reattach the labrum and create a capsular shift. These steps were also repeated with at least 3 anchors.

Postoperatively, all patients followed a standardized rehabilitation protocol for arthroscopic shoulder stabilization at our department. The arm was immobilized in a neutral position in a sling for 6 weeks, with passive mobilization exercises limited to 60° of flexion and abduction and 0° of external rotation during this period. After 6 weeks, active assisted exercises to 90° of flexion and abduction and 0° of external rotation were initiated and active range of motion and exercises were allowed.

### Radiological and Clinical Evaluations

Preoperative glenoid defects were retrospectively assessed using the PICO method in the en face view of computed tomography.^4^ A best-fit circle was applied to estimate the area of the original glenoid, and the defect size was calculated as a percentage.

The on-track off-track method, described by Gyftopoulos et al,^14^ was used to evaluate Hill-Sachs lesions on magnetic resonance imaging. In patients with no available computed tomography or magnetic resonance imaging scans (85 shoulders), intraoperative arthroscopic images and operative reports were used to assess the glenoid defect and glenoid track. Glenoid defects >20% and off-track Hill-Sachs lesions were considered indications for bony reconstruction, and therefore, these patients were excluded from the study.

The primary outcome of the present study was recurrent instability, defined as a redislocation or subluxation. The secondary outcomes were shoulder function and sports activity. The in-person follow-up examination and review of electronic medical records were conducted by a single observer and included a medical history of shoulder instability, a standard clinical assessment of both shoulders, and questionnaires assessing shoulder outcomes. The number, date, and cause of preoperative and postoperative subluxations and dislocations; revision surgery; and return to sports were recorded. To assess sports activity, all types of sports; the sports activity level according to Valderrabano et al^32^ preoperatively, postoperatively (highest level after surgery), and at follow-up (level at the time of follow-up examination); and the recovery of athletic activity according to Rhee et al^28^ were noted. The physical examination included an assessment of range of motion, the anterior apprehension test, the Gagey test^12^ to evaluate shoulder hyperlaxity on the contralateral side, an assessment of scapular dyskinesis, and an assessment of strength using an isometric strength measurement device (IsoForceControl EVO2 dynamometer; Medical Device Solutions). The apprehension test finding was considered positive only when patients reported a feeling of subjective instability. Scapular dyskinesis was rated based on the classification system described by Kibler et al^20^ and further assessed using the scapular assistance test, scapular retraction test, dynamic scapular dyskinesis test, lateral scapular slide test, and SICK Scapula Rating Scale.^5^

Functional outcomes were evaluated using the following outcome measures: Subjective Shoulder Value (SSV),^13^ visual analog scale (VAS) for pain at rest and during movements, Constant score,^8^ Western Ontario Shoulder Instability Index (WOSI),^21^ and Rowe score.^30^ Secondary outcomes for patients with revision surgery were documented and evaluated separately. Potential risk factors were assessed with respective subgroup analyses, including sex, age at surgery, hand dominance, sports activity, hyperlaxity, bony defects, number of preoperative dislocations, time from first dislocation to surgery, and number and type of anchors.

### Statistical Analysis

Statistical analysis was performed with SPSS Statistics software (Version 29; IBM), and *P* < .05 was considered as statistically significant. The Kolmogorov-Smirnov test was used to examine data for a normal distribution. Patient characteristics were reported using descriptive statistics. Categorical variables were presented as the frequency and percentage. Continuous variables were presented as the mean ± standard deviation when normally distributed or as the median (interquartile range [IQR]) when nonnormally distributed. The unpaired *t* test (for normally distributed data) and the Mann-Whitney *U* test (for nonnormally distributed data) were performed to compare continuous variables between groups. The paired 2-sample *t* test was used to compare continuous variables between the preoperative and postoperative time points. For dichotomous data, the chi-square test was used. The correlation between continuous variables and outcome scores was assessed using the Spearman correlation coefficient. Kaplan-Meier survival analysis was performed to assess the time from surgery to recurrence, and the log-rank test was used to compare survival times. Cox regression was used to analyze hazard ratios. Binary logistic regression was performed to analyze the influence of potential risk factors on recurrent instability and to calculate the odds ratio with 95% confidence interval (CI).

## Results

Patient and surgical characteristics are presented in Table 1.

**Table 1:** Patient and Surgical Characteristics*^a^*

	Value (n = 182 Shoulders)
Follow-up period, mean ± SD, y	13.8 ± 2.8
Male sex	152 (83.5)
Dominant arm affected	86 (47.3)
Shoulder hyperlaxity	45 (24.7)
Beighton score	
≤4	167 (91.8)
>4	15 (8.2)
No. of preoperative dislocations	3 (1-7)
1	58 (31.9)
>1	124 (68.1)
Age at surgery, y	27 (22-36)
Time to surgery, mo	12.2 (1.2-60.3)
Glenoid defect,*^b^* %	2.1 (0.0-5.4)
Type of suture anchors*^c^*	
All-suture	17 (9.8)
Hard-body	156 (90.2)
No. of suture anchors*^c^*	3 (3-3)
3	136 (78.6)
>3	37 (21.4)

a Data are expressed as n (%) or median (interquartile range) unless otherwise specified.

b Data were available for 97 shoulders.

c Data were unavailable for 9 shoulders.

### Recurrent Instability

At follow-up, recurrent instability was observed in 38 of 182 shoulders (20.9%), including 25 shoulders (13.7%) with redislocations and 13 shoulders (7.1%) with subluxations. The median number of recurrences was 2 (IQR, 1-5) until last follow-up. Of the 38 shoulders with recurrent instability, 31 (81.6%) experienced recurrence after a new traumatic event, such as a fall on the shoulder.

**Figure 2.**
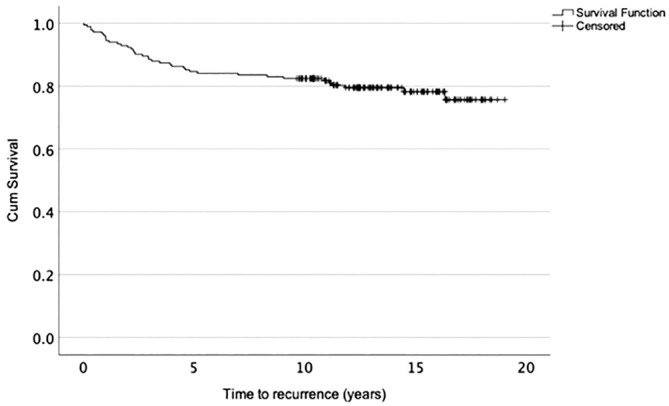
Kaplan-Meier survival curve of recurrence rate.

The median time from surgery to recurrence was 2.8 years (IQR, 1.0-5.6 years). In 13 shoulders (34.2%), recurrence occurred within the first 2 years after surgery, in 15 shoulders (39.5%) between 2 and 5 years, in 4 shoulders (10.5%) between 5 and 10 years, and in 6 shoulders (15.8%) after 10 years postoperatively. Of the 7 cases of atraumatic recurrence, 5 (71.4%) occurred within the first 2.5 years, 1 after 5.2 years, and 1 after 14.5 years. The 10-year survival rate without recurrent instability was 82.4% (95% CI, 77.1%-87.7%) (Figure 2).

The overall revision rate was 7.7%, with all revision procedures performed because of failure. Revision surgery for instability was performed in 36.8% of shoulders (n = 14) with recurrence: arthroscopic Bankart repair in 5 shoulders (2.7%), the Latarjet procedure in 8 shoulders (4.4%), and iliac crest bone graft transfer in 1 patient (0.5%). The other patients were treated nonoperatively or declined reoperations.

### Risk Factors for Recurrent Instability

The age of the patient at the time of surgery was a risk factor associated with recurrent instability. The recurrence rate was highest in patients aged <20 years (36.4%) and declined significantly with increasing age (*P* = .029). For patients aged 20 to 29 years, the recurrence rate decreased to 25.6%; in patients aged 30 to 39 years, it further dropped to 12.5%; and for patients aged ≥40 years, the rate was the lowest at 8.8%.

The hazard ratio for recurrent instability between patients aged ≤20 years and those aged >20 years was 2.4 (95% CI, 1.2-4.9; *P* = .01). Patients who underwent surgery after the first-time dislocation had a lower recurrence rate (8/58 [13.8%]) compared with those with >1 dislocation before surgery (30/124 [24.2%]), although this difference was not statistically significant (*P* = .108). Logistic regression analysis demonstrated that young age at the time of surgery was the only risk factor associated with a significantly higher risk of failure (*P* = .001) (Table 2).

**Table 2:** Risk Factors for Recurrent Instability*^a^*

	Recurrence, n (%)	*P* Value*^b^*
Sex		.534
Male (n = 152)	33 (21.7)	
Female (n = 30)	5 (16.7)	
Age at surgery		**.007**
≤20 y (n = 31)	12 (38.7)	
>20 y (n = 151)	26 (17.2)	
Affected arm		.703
Dominant (n = 86)	19 (22.1)	
Nondominant (n = 96)	19 (19.8)	
Type of sports before surgery		.453
Overhead or collision (n = 130)	29 (22.3)	
No overhead or collision (n = 52)	9 (17.3)	
Sports level		.766
Competitive (n = 26)	6 (23.1)	
Not competitive (n = 156)	32 (20.5)	
Shoulder hyperlaxity		.798
Yes (n = 45)	10 (22.2)	
No (n = 137)	28 (20.4)	
Type of suture anchors*^c^*		.395
All-suture (n = 17)	5 (29.4)	
Hard-body (n = 156)	32 (20.5)	
	Odds Ratio (95% CI)	*P* Value*^d^*
Age at surgery, y	0.925 (0.881-0.970)	**.001**
No. of preoperative dislocations	0.998 (0.990-1.007)	.709
Glenoid defect,*^e^* %	0.914 (0.771-1.083)	.297
Time to surgery, mo	0.999 (0.995-1.003)	.538
No. of suture anchors*^c^*	0.945 (0.421-2.119)	.890

a Bold *P* values are statistically significant (*P* < .05).

b The chi-square test was used.

c Data were unavailable for 9 shoulders.

d Binary logistic regression was used.

e Data were available for 97 shoulders.

### Shoulder Function

The apprehension test finding was positive in 17.6% (32/182) of all shoulders. Of the shoulders without revision surgery, the apprehension test finding was positive in 12.5% (18/144) without recurrent instability and in 54.2% (13/24) with recurrence (*P* < .001). Of the shoulders with revision surgery, the apprehension test finding was positive in 7.1% (1/14).

The shoulder outcome scores for patients without revision surgery (168 shoulders) are shown in Table 3. Patients without recurrent instability had significantly better scores on shoulder outcome measures and apprehension test results compared with patients with recurrence. Additionally, a negative correlation was observed between the time from the first dislocation to surgery and the SSV score (ρ = −0.174; *P* = .025) and the Rowe score (ρ = −0.214; *P* = .006) as well as a positive correlation between the time from the first dislocation to surgery and the WOSI score (ρ = 0.182; *P* = .019).

**Table 3:** Shoulder Outcome Scores of Patients Without Revision Surgery*^a^*

	No Recurrence (n = 144)	Recurrence (n = 24)	*P* Value
SSV	90 (80-100)	80 (70-90)	**<.001**
VAS for pain at rest	00 (0-0)	00 (0-0)	.204
VAS for pain during movements	00 (0-1)	1 (0-5)	**.016**
Constant score	92 (86-96)	89 (81-92)	**.011**
WOSI	30 (25-53)	48 (32-103)	**.001**
Rowe score	95 (80-100)	45 (40-50)	**<.001**
SICK Scapula Rating Scale	2 (1-3)	2 (1-3)	.369
Scapular dyskinesis, n (%)*^b^*	41 (36.3)	10 (52.6)	.176
Apprehension, n (%)	18 (12.5)	13 (54.2)	**<.001**

a Data are expressed as median (interquartile range) unless otherwise specified. Bold *P* values are statistically significant (*P* < .05). SSV, Subjective Shoulder Value; VAS, visual analog scale; WOSI, Western Ontario Shoulder Instability Index.

b Data were available for 132 shoulders.

### Sports Activity Level and Return to Sports

The sports activity levels according to Valderrabano et al^32^ preoperatively, postoperatively (highest level after surgery), and at follow-up (level at the time of follow-up examination) for all patients without revision surgery (168 shoulders) are shown in Table 4. Preoperatively, the overall sports activity rate was 94.0%, and the mean sports activity level was 2.36 ± 1.00, which corresponds to a normal sports activity level of 1 to 5 h/wk. Postoperatively, the sports activity rate remained at 94.0%, and the mean sports activity level was similar to the preoperative level. No statistically significant difference was found between the sports activity levels preoperatively and postoperatively (*P* = .569).

**Table 4:** Sports Activity Level*^a^*

	Preoperative	Postoperative (highest level after surgery)	At Follow-up (level at the time of follow-up)
0 (no sports)	10 (6.0)	10 (6.0)	23 (13.7)
1 (moderate: <1 h/wk)	11 (6.5)	8 (4.8)	18 (10.7)
2 (normal: 1-5 h/wk)	80 (47.6)	83 (49.4)	84 (50.0)
3 (high: >5 h/wk)	43 (25.6)	52 (30.9)	41 (24.4)
4 (competitive sports)	24 (14.3)	15 (8.9)	2 (1.2)
Mean ± SD	2.36 ± 1.00	2.32 ± 0.92	1.89 ± 0.97

a Data are expressed as n (%) unless otherwise specified.

The recovery of athletic activity was rated according to Rhee et al^28^ (Table 5). The patients reported a median return-to-sports level of 1.5 (IQR, 1-3), equivalent to 90% to 100% of their previous athletic activity. In total, 84 of 168 shoulders (50.0%) returned to 100% of their previous activity. A statistically significant difference in the recovery of sports was observed between patients with and without recurrent instability.

**Table 5:** Recovery of Athletic Activity*^a^*

	No Recurrence (n = 144)	Recurrence (n = 24)	*P* Value
1 (full recovery: 100%)	76 (52.8)	8 (33.3)	
2 (≥90%)	29 (20.1)	4 (16.7)	
3 (≥70%)	26 (18.1)	9 (37.5)	
4 (≥50%)	10 (6.9)	2 (8.3)	
5 (no return to athletic activity or difficulty in daily activities)	3 (2.1)	1 (4.2)	
Median (IQR)	1.0 (1-3)	2.5 (1-3)	**.043**

a Data are expressed as n (%) unless otherwise specified. Bold *P* values are statistically significant (*P* < .05). IQR, interquartile range.

### Revision Surgery

The shoulder outcome scores for the patients who underwent revision surgery are presented in Table 6. No statistically significant differences were observed between patients who underwent revision surgery for recurrence and those without recurrent instability, except for the VAS for pain at rest and during movements.

**Table 6:** Shoulder Outcome Scores of Patients Without Recurrence and Patients With Revision Surgery*^a^*

	No Recurrence Without Revision (n = 144)	Recurrence With Revision (n = 14)	*P* Value
SSV	90 (80-100)	90 (76-95)	.196
VAS for pain at rest	00 (0-0)	00 (0-1)	**.037**
VAS for pain during movements	00 (0-1)	3 (0-7)	**.002**
Constant score	92 (86-96)	87 (67-95)	.164
WOSI	30 (25-53)	48 (25-88)	.355
Rowe score	95 (80-100)	93 (71-96)	.109
SICK Scapula Rating Scale	2 (1-3)	3 (0-5)	.569

a Data are expressed as median (interquartile range). Bold *P* values are statistically significant (*P* < .05). SSV, Subjective Shoulder Value; VAS, visual analog scale; WOSI, Western Ontario Shoulder Instability Index.

## Discussion

Arthroscopic Bankart repair using a contemporary technique with a minimum of 3 suture anchors in an appropriately selected patient cohort showed good to excellent results in shoulder function and return to sports at a minimum follow-up of 10 years. The recurrence rate of 20.9% is consistent with rates reported in the literature.

Previous studies have reported the long-term results of arthroscopic Bankart repair.^25^ However, techniques and devices have evolved over the past decades from transglenoid sutures^6^ and bioabsorbable tacks^35^ to modern techniques with suture anchors.^23,36^ Earlier arthroscopic stabilization techniques resulted in a higher rate of redislocations and complications and have consequently been discontinued in clinical practice.^11,23^ Murphy et al^25^ conducted a systematic review of studies reporting on the long-term outcomes of arthroscopic Bankart repair at a minimum of 10 years’ follow-up and found an overall recurrence rate of 31.2%. This high failure rate might be because previous studies with a minimum follow-up of 10 years included patients stabilized with older techniques or <3 anchors.^1,7,10,19,26,27,38^ Moreover, the significance of bone loss on both the humeral and glenoid sides and the importance of preoperative assessments through imaging became more evident with the introduction of the glenoid track concept in 2007 by Yamamoto et al.^37^ A systematic review by Leroux et al^22^ demonstrated that the failure rate after arthroscopic Bankart repair in contact or collision athletes decreased from 17.8% to 7.9% when evidence-based surgical indications and techniques were used.

In this study, we used a contemporary technique with at least 3 suture anchors placed between the 3- and 6-o’clock positions. The recurrence of a dislocation or a subluxation after surgery was considered as failure, and apprehension was assessed separately. Our recurrence rate of 20.9% is the lowest reported failure rate of arthroscopic Bankart repair at a minimum follow-up of 10 years, except for a study by Aboalata et al,^1^ which reported a recurrence rate of 18.18%. However, in their study, subjectively reported subluxations without clinical signs of instability were not counted as recurrence, whereas in our study, all subluxations were considered as failures because dislocations and subluxations can occur without a persisting feeling of instability or apprehension.^31^ If subjectively reported subluxations after surgery were not considered as recurrence, 8 of 38 cases of recurrence in our study would not count as failures, and the recurrence rate would decrease to 16.5%. Looking at our recurrence pattern, our results indicate that short-term studies with a minimum follow-up of 2 years are not enough to estimate the actual failure rate, as suggested by Rossi et al.^29^

Patients aged ≤20 years were particularly at risk for instability recurrence. This finding is consistent with previously reported risk factors in the literature.^1,2,34^ Balg and Boileau^2^ identified age <20 years as a risk factor for recurrent shoulder instability and integrated this factor into the Instability Severity Index Score in 2007. Subsequent clinical studies by Aboalata et al^1^ and Vermeulen et al^34^ confirmed the significance of this observation and demonstrated a significant decrease in the recurrence rate with the advancement of age. One possible explanation is that younger patients tend to be more physically active and participate more frequently in high-risk sports, making them more susceptible to redislocations.

Furthermore, patients who underwent arthroscopic Bankart repair after a first-time dislocation showed a lower recurrence rate (13.8%) compared with patients with >1 dislocation before surgery (24.2%). Although this difference was statistically not significant in our study, Marshall et al^24^ demonstrated a significantly higher recurrence rate in the recurrent dislocation group compared with the first-time dislocation group (62% vs 29%, respectively) at a mean follow-up of 52 months in 121 patients. However, it should be noted that some patients in the first-time dislocation group likely would have become stable even if they had been treated nonoperatively. In a study by Hovelius et al,^16^ 245 patients with a primary anterior shoulder dislocation were observed over a 10-year period, and in 52% of the patients, no additional dislocation occurred after the first-time dislocation. While the time to surgery did not correlate with the recurrence rate in our study, a shorter interval between the first dislocation and surgery was associated with improved clinical outcomes. We therefore believe that patients with a high risk of redislocations should be considered for early surgical treatment to lower the risk of postoperative recurrent instability and to achieve better clinical scores.

Our study found no association between the size of the glenoid defect and recurrent instability most likely because of the exclusion of patients with significant bony defects. Similarly, long-term studies by van der Linde et al^33^ and Vermeulen et al^34^ also reported no association between glenoid defects and recurrent instability.

As expected, the patients with recurrent instability reported poorer shoulder outcome scores for the SSV, VAS for pain, Constant score, WOSI, and Rowe score compared with patients without recurrence. However, no significant difference in shoulder function was found between patients who underwent revision surgery and those who did not undergo revision surgery. It appears that revision surgery may improve shoulder function in patients with recurrence to a level comparable with those without recurrence.

This study has several limitations. Because of the long follow-up period, only 182 of the 321 included shoulders (56.7%) were available for the follow-up examination, resulting in a high rate of loss to follow-up and potential attrition bias. Furthermore, because of the retrospective design of this study, no preoperative scores were available for comparison with the postoperative outcome scores. Additionally, the group sizes for the subgroup analysis were unequal, which could introduce statistical bias. Although medical records were thoroughly reviewed, some data regarding the number of dislocations, bony defects, and type of anchors used were missing and thus subject to recall bias. Further, no radiographs were obtained to assess potential osteoarthritis at last follow-up.

## Conclusion

Arthroscopic Bankart repair resulted in a relatively high recurrence rate, despite the use of a contemporary technique, particularly in patients with >1 dislocation before surgery. While younger age and number of preoperative dislocations were potential risk factors for recurrence, a shorter interval between the first dislocation and surgery was associated with improved clinical outcomes. Therefore, patients with a high risk of redislocations should be considered for early soft tissue stabilization, while additional procedures such as remplissage should be performed for those with nonmodifiable high-risk factors.
